# Village-based spatio-temporal cluster analysis of the schistosomiasis risk in the Poyang Lake Region, China

**DOI:** 10.1186/s13071-017-2059-y

**Published:** 2017-03-08

**Authors:** Congcong Xia, Robert Bergquist, Henry Lynn, Fei Hu, Dandan Lin, Yuwan Hao, Shizhu Li, Yi Hu, Zhijie Zhang

**Affiliations:** 1 Department of Epidemiology and Biostatistics, School of Public Health, Fudan University, Shanghai, 200032 China; 2 Key Laboratory of Public Health Safety, Ministry of Education, Shanghai, 200032 China; 3 Laboratory for Spatial Analysis and Modeling, School of Public Health, Fudan University, Shanghai, 200032 China; 4 Collaborative Innovation Center of Social Risks Governance in Health, School of Public Health, Fudan University, Shanghai, 200032 China; 5 Ingerod, Brastad, Sweden; 6 Jiangxi Institute of Schistosomiasis Prevention and Control, Nanchang, 330000 China; 7 National Institute of Parasitic Diseases, Chinese Center for Disease Control and Prevention, Key Laboratory of Parasite and Vector Biology, Ministry of Health, Shanghai, 200032 China

**Keywords:** Schistosomiasis, Spatio-temporal, Poyang Lake Region, China

## Abstract

**Background:**

The Poyang Lake Region, one of the major epidemic sites of schistosomiasis in China, remains a severe challenge. To improve our understanding of the current endemic status of schistosomiasis and to better control the transmission of the disease in the Poyang Lake Region, it is important to analyse the clustering pattern of schistosomiasis and detect the hotspots of transmission risk.

**Results:**

Based on annual surveillance data, at the village level in this region from 2009 to 2014, spatial and temporal cluster analyses were conducted to assess the pattern of schistosomiasis infection risk among humans through purely spatial (Local Moran’s *I*, Kulldorff and Flexible scan statistic) and space-time scan statistics (Kulldorff). A dramatic decline was found in the infection rate during the study period, which was shown to be maintained at a low level. The number of spatial clusters declined over time and were concentrated in counties around Poyang Lake, including Yugan, Yongxiu, Nanchang, Xingzi, Xinjian, De’an as well as Pengze, situated along the Yangtze River and the most serious area found in this study. Space-time analysis revealed that the clustering time frame appeared between 2009 and 2011 and the most likely cluster with the widest range was particularly concentrated in Pengze County.

**Conclusions:**

This study detected areas at high risk for schistosomiasis both in space and time at the village level from 2009 to 2014 in Poyang Lake Region. The high-risk areas are now more concentrated and mainly distributed at the river inflows Poyang Lake and along Yangtze River in Pengze County. It was assumed that the water projects including reservoirs and a recently breached dyke in this area were partly to blame. This study points out that attempts to reduce the negative effects of water projects in China should focus on the Poyang Lake Region.

## Background

Intestinal schistosomiasis caused by infection with *S. japonicum*, a water-borne parasitic disease, has brought about significant economic and public health concerns in south-east Asia [1, 2] with a history of more than 2100 years in China [3]. Epidemiological surveys showed that approximately 100 million people were at risk, with up to 12 million being infected with schistosomiasis, at the founding of the People’s Republic of China in 1994 [4]. Over the past 60 years, China has been taking effective steps towards eliminating *Schistosoma japonicum* [5]. During 1992–2001, the World Bank committed a loan of 71 million US dollars that was expanded by matching funds at the same level from the Government of China for schistosomiasis control mainly based on chemotherapy and snail control. These measures reduced the prevalence by over 50% [6], while the further emphasis of control activities through a revised strategy was initiated from 2005 based on new diagnostic techniques, replacement of cows with machines and a boost of health education made further significant progress in controlling this disease [6]. According to the latest report in 2014, the number of people infected was estimated to be 115,614, which was a decrease of 83.4% since 2000 [7]. Meanwhile, of the 12 endemic provinces located along the Yangtze River, five (Shanghai, Zhejiang, Fujian, Guangdong, Guangxi) reached transmission interruption criterion, and four (Sichuan, Yunnan, Jiangsu, Hubei) reached transmission control criterion [8].

Despite the remarkable success achieved in schistosomiasis control in China through the measures mentioned, schistosomiasis remains a serious problem in the lake and marshland regions, especially along the middle of the Yangtze River basin (including both Dongting and Poyang Lakes). Contributing factors are the parasite’s complicated transmission process based on a snail intermediate host, anthropogenic environment transformation and regional climate change [5, 9, 10]. It has been estimated that people infected with *S. japonicum* in swamp and lake areas account for approximately 86% of the total number in the whole country [11]. Poyang Lake Region, which includes the largest freshwater lake in China, constitutes the largest continuous area endemic for schistosomiasis in the country and was historically reported with 2.5 million people at risk for infection and 340,000 infected [12].

The year 2016 represents the aftermath of the preceding year’s ultra-strong El Nino [13] and, the climate has been extraordinarily complex with 27 rainstorms since the flooding season according to the National Climate Center in China [14]. The water level at the Hukou hydrometric station in Poyang Lake was 1.18 m higher than last year at the corresponding period, and flooding disasters occurred in Jiangxi Province including Poyang, Duchang, Yongxiu Counties. Flooding impacts the schistosomiasis situation negatively and, indeed, the number of acute cases of the disease have been markedly higher in years characterised by flooding [15]. Investigation of cluster patterns is therefore distinctly important for implementing control measures during and after floods.

It is widely acknowledged that the epidemiology of *S. japonicum* infections has a particular spatial characteristic in China because it depends on the presence of a sole intermediate host snail, *Oncomelania hupensis*, whose specific climatic and environmental conditions for reproduction governs the distribution of schistosomiasis [16–18]. Recent advancement of specific spatial analysis tools, such as SaTScan and FlexScan facilitate the investigation of schistosomiasis’ epidemiological patterns in space and time at scales varying from local to regional [17]. In previous studies [6, 19], spatial clusters of schistosomiasis have ever been detected in endemic areas, but they only made use of traditional spatial scan statistics as SaTScan spatial scan and Local Moran’s I. Considering that the extent of these clusters are commonly irregular, we also applied a new spatial scan statistic method (Flexible scan statistic).

In this study, *S. japonicum* prevalence data at the village level from 2009 to 2014 in Jiangxi Province, China, was evaluated to analyse the spatial and temporal clustering of schistosomiasis, using the three methods mentioned above to help improve our understanding of the current endemic status in the Poyang Lake Region.

## Methods

### Study area

**Fig. 1:**
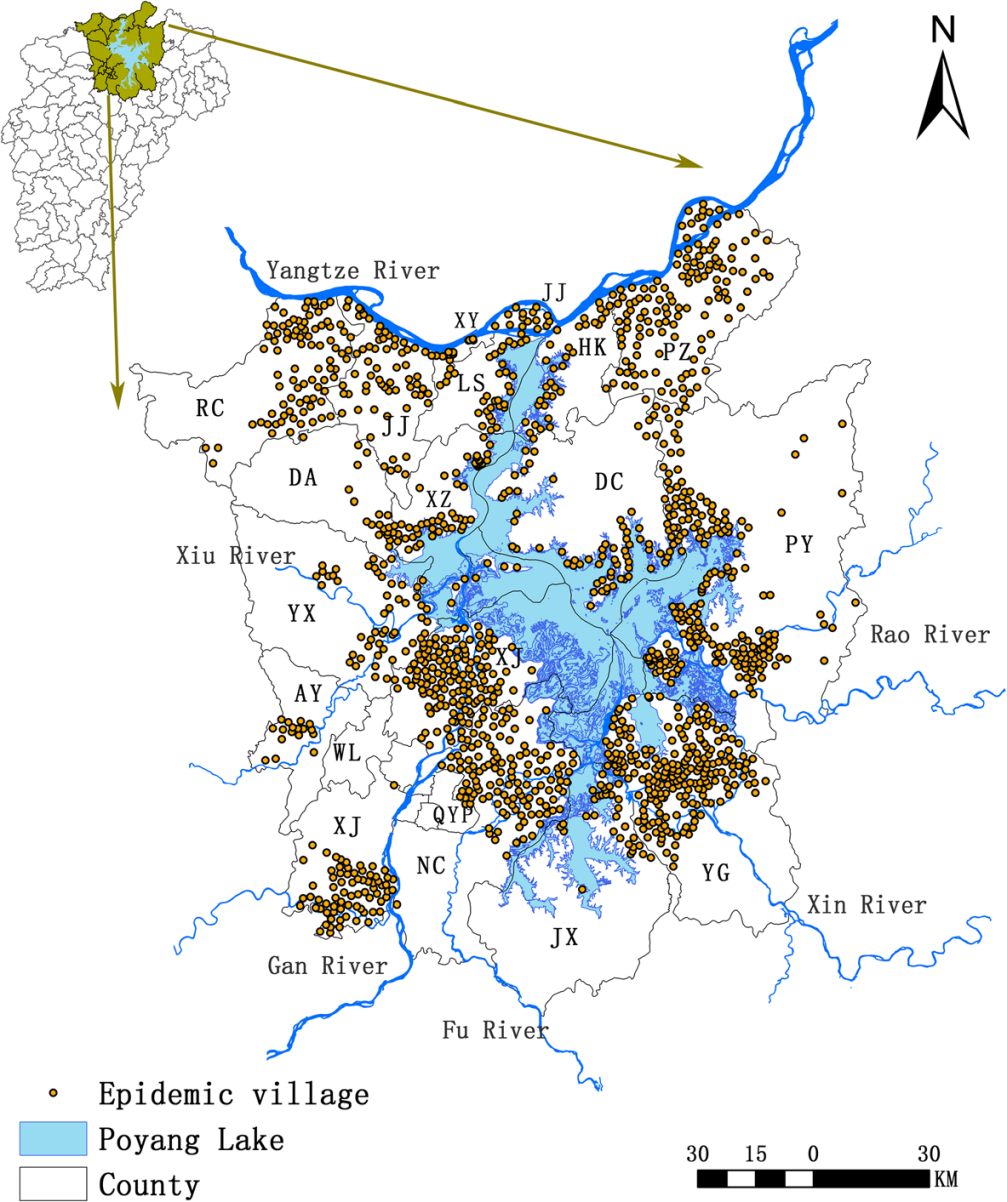
Location of the study area and the spatial distribution of survey data. Poyang Lake is situated in the northern part of Jiangxi Province and near the southern bank of the middle and lower reaches of River Yangtze. This study covers the core region surrounding the lake with about 18 endemic counties including 1374 endemic administrative villages. *Abbreviations*: AY, Anyi; DA, De’an; DC, Duchang; HK, Hukou; JJ, Jiujiang; JX, Jinxian; LS, Lushan; NC, Nanchang; PY, Poyang; PZ, Pengze; QYP, Qingyunpu; RC, Ruichang; WL, Wanli; XJ, Xinjian; XY, Xunyang; XZ, Xingzi; YG, Yugan; YX, Yongxiu

Poyang Lake, the largest freshwater lake in China, is situated in the northern part of Jiangxi Province and near the southern bank of the middle and lower reaches of River Yangtze (Fig. 1). Five main tributaries, rivers Gan, Fu, Xiu, Rao and Xin, flow into Poyang Lake. Poyang Lake is a typical seasonal water-land transition lake with dramatic intra-annual fluctuation of the water level (historically ranging from 7.1 m in the winter dry season to 19.0 m in the summer rainy season) [20], leading to a variation of lake coverage from around 500 km^2^ in the winter to a peak of approximately 4125 km^2^ in the summer [21]. During flooding, water recharges from River Yangtze, while it is deficient at the other extreme of the season, which generates flood plains from April to September and a landscape of marshlands from November to early March [12]. These dramatic variations of the water level along with the humid, subtropical monsoon in Jiangxi Province govern the distribution of the parasite’s intermediate snail host *O. hupensis* [22].

This study covered the core region surrounding the lake with about 18 endemic counties, including 1374 administrative villages. The latitude and longitude coordinates of each village were obtained by a handheld Global Position System (GPS) instrument (MobileMapper10, Thales Navigation, Paris, France).

### Calculation of infection rates

Village-level data on schistosomiasis endemicity in human from 2009 to 2014 were collected and processed. The database was setup under annually conventional surveillance in each endemic village of Jiangxi Province.

A two-step diagnostic approach confirmed human infection with schistosomiasis. Residents in endemic villages were first screened by the indirect haemagglutination (IHA) serology test and those found to be positive were further tested by the faecal Kato-Katz parasitological test, currently the gold standard for diagnosis of *S. japonicum* [23].

In this study, one indicator was calculated to reflect the endemic risk with the following formula:

$$ \mathrm{Human}\ \mathrm{in}\mathrm{fection}\ \mathrm{rate}\ \left(1/100,000\right)=\frac{\mathrm{Number}\ \mathrm{of}\ \mathrm{positive}\ \mathrm{cases}\kern0.5em \mathrm{by}\ \mathrm{Kato}\hbox{-} \mathrm{Katz}\ }{\mathrm{Number}\ \mathrm{of}\ \mathrm{in}\mathrm{dividuals}\ \mathrm{participating}\ \mathrm{in}\ \mathrm{the}\ \mathrm{survey}}\times 100,000/100,000 $$

### Statistical analysis

First, a descriptive statistical analysis for each year was carried out to compare changes in the infection rate of schistosomiasis during the epidemics over time. Secondly, Anselin’s Local Moran’s *I* [24], a widely used spatial autocorrelation analysis, was implemented in the ArcGIS software, version 10.0 (ESRI. Redlands, CA, USA) to detect the significant high-high and high-low villages as hotspots. To describe the neighbouring relation between villages, Thiessen polygons (ESRI) were generated. If two villages shared a common border, the spatial relationship was considered so that the weight element equalled 1 for one of the villages and 0 for the other. In the end, the significant areas of high rates detected were converted back to points for distinct visualisation.

Thirdly, purely spatial cluster analysis annually was performed by Kulldorff’s spatial scan statistic using SaTScan software, version 9.3 (http://www.satscan.org/) to detect clusters by a circular window. The detecting window was centred on each of several possible grid points positioned throughout the study region with a radius that could be varying continuously in size from zero to some upper limit specified by the user. The maximum spatial cluster size was set to be 50% of the population at risk. We also used the flexible scan statistic implemented by the FleXScan software, version 3.1.2 (https://sites.google.com/site/flexscansoftware/), which imposed an irregularly shaped window to detect clusters within relatively small neighbourhoods of each region. The maximum spatial cluster size was set to be 15 census areas (default = 15) in this study.

Fourthly, the frequency of spatial cluster occurrence was calculated for each village in the study area and expressed as the number of years during 2009 to 2014 of each high-risk village contributed to a cluster. A ‘high-risk village’ was defined as one detected in a cluster by two of the statistical methods applied, while at the same time, one of which found in a cluster by all methods mentioned above was recognised as a ‘strong evidence high-risk village’.

Finally, the space-time scan statistic was defined as a cylindrical window with a circular geographical base and with the height corresponding to time (performed using SaTScan software). Not only the maximum spatial cluster size was set at 50% of the population at risk as the pure Kulldorff’s spatial scan statistic, but the maximum temporal cluster size was also set at 50% of the study period in the retrospective space-time analysis of the six-year period in this study.

The relative risk (RR) and the log-likelihood ratio of each potential cluster both by SaTScan and FlexScan were calculated based on observed and expected infection rate inside and outside the scan window. All the cluster scan statistics used in this study were performed based on Poisson model, and a *P*-value of the test was calculated with a likelihood ratio test statistic using 999 random simulations Monte Carlo replications under the null hypothesis of the null distribution. Statistically significant results were considered as *P <* 0.05. The final results were imported into ArcGIS 10.0 (ESRI) and generated to visualised maps for risk cluster analysis.

## Results

### Variation of the endemic status of schistosomiasis in Poyang Lake Region

As shown in Table 1, the number of endemic villages fluctuated smoothly in the range from 903 to 998 before 2012 and reached 1067 in 2014. The number of people at risk of infection increased continuously, but the number of infection cases each year showed a tendency of prominently continuous decline except for a slight rebound in 2010. The number of infection cases increased slightly from 1383 in 2009 to 1584 in 2010, then decreased substantially to 95 in 2014. The crude infection rate of human schistosomiasis showed a pronounced decrease year by year from 193.8/100,000 in 2009 to 8.8/100,000 in 2014, thus experiencing a decline of 95.5%.

**Table 1:** Summary data for schistosomiasis in the Poyang Lake Region, China

Year	Number of endemic villages	Endemic population	Number of human cases	Crude infection rate per 100,000 inhabitants
2009	903	714,143	1383	193.8
2010	987	869,483	1584	182.3
2011	945	929,149	1054	113.5
2012	998	967,328	304	31.4
2013	978	984,367	386	39.2
2014	1067	1,082,355	95	8.8

### Pure spatial cluster analysis of the schistosomiasis infection rate in humans

**Fig. 2:**
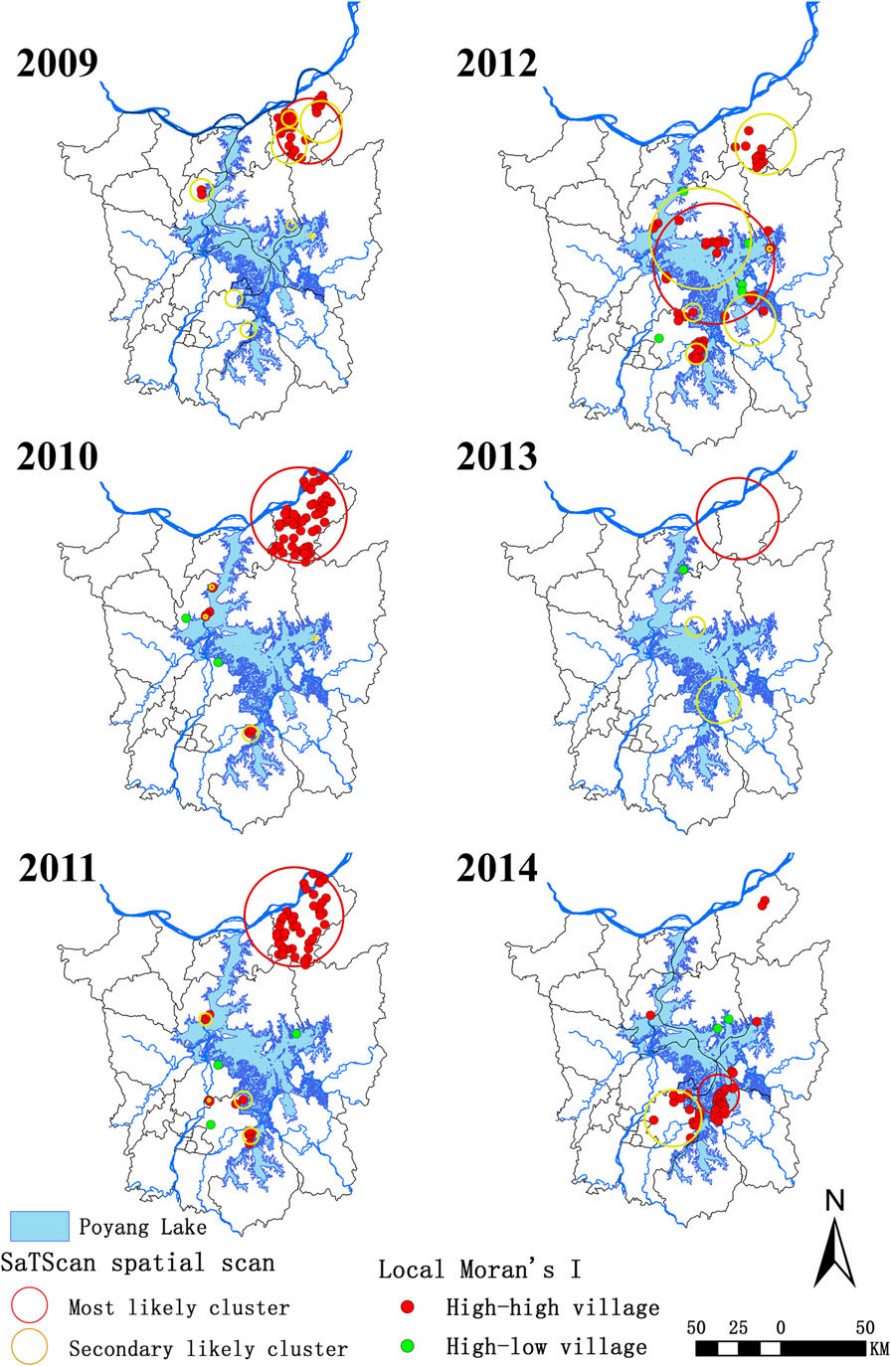
Annual number and distribution of spatial clusters of schistosomiasis cases identified in the period 2009–2014. Each panel shows the results of both methods: Local Moran’s *I* (Anselin) and SaTScan (Kulldorff). During 2009 to 2014, 167 high-risk counties were identified by Local Moran’s *I* with an annual number ranging from 1 (in 2013) to 63 (in 2010). The number of clusters defined by SaTScan decreased from 15 in 2009 to 3 in 2014 with an obvious tendency to transfer the focus to the South

As shown in Fig. 2, spatial clusters for each year from 2009 to 2014 were detected by both Kulldorff’s spatial scan statistic (SaTScan) and Local Moran’s *I* (Anselin). During 2009 to 2014, the Local Moran’s *I* identified 167 high-risk counties, with an annual number ranging from 1 (in 2013) to 63 (in 2010). SaTScan spatial scan clusters were consistent with those detected by Local Moran’s *I*. Figure 2 and Table 2 show that the number of clusters detected by SaTScan method decreased from 15 in 2009 to 3 in 2014. The annual most likely cluster extended first from 2009 to 2012, then shrank back with endemic villages ranging from 25 (in 2014) to 150 (in 2012). Considering the changing pattern of spatial distribution among the clusters detected by SaTScan, an obvious tendency of a transfer to the South was observed. Most likely clusters were mainly concentrated in Pengze County from 2009 to 2013 except in 2012 when they shifted to the central section of Poyang Lake across several counties including Duchang, Poyang, Yugan and Xinjian. Most likely clusters were also seen in the southern part of Poyang Lake, mainly in Yugan County in 2014. There were several slight, likely secondary clusters surrounding Poyang Lake annually in the period 2009 to 2011. While the extent of clustering tended to enlarge after that and mainly become located in Pengze County, Yugan County and Nanchang County. Also, a larger cluster distributed across several counties in the middle part of Poyang Lake was observed in 2012.

**Table 2:** Spatial analysis scanning for clusters with high rates using the discrete Poisson model by Kulldorff’s spatial scan statistic

Year	No. of clusters	Most likely cluster						
		No. of villages	Radius (km)	No. of cases	Expected no. of cases	RR^a^	LLR^b^	*P*-value
2009	15	51	19.14	626	82.75	12.99	857.21	< 0.001
2010	11	85	27.78	887	157.08	11.56	1036.11	< 0.001
2011	8	91	28.72	598	122.57	9.97	622.09	< 0.001
2012	8	150	33.63	111	45.74	3.25	42.19	< 0.001
2013	3	79	23.65	185	32.81	9.91	206.70	< 0.001
2014	3	25	12.06	31	2.24	20.06	57.70	< 0.001

^a^Relative risk

^b^Log likelihood ratio

**Fig. 3:**
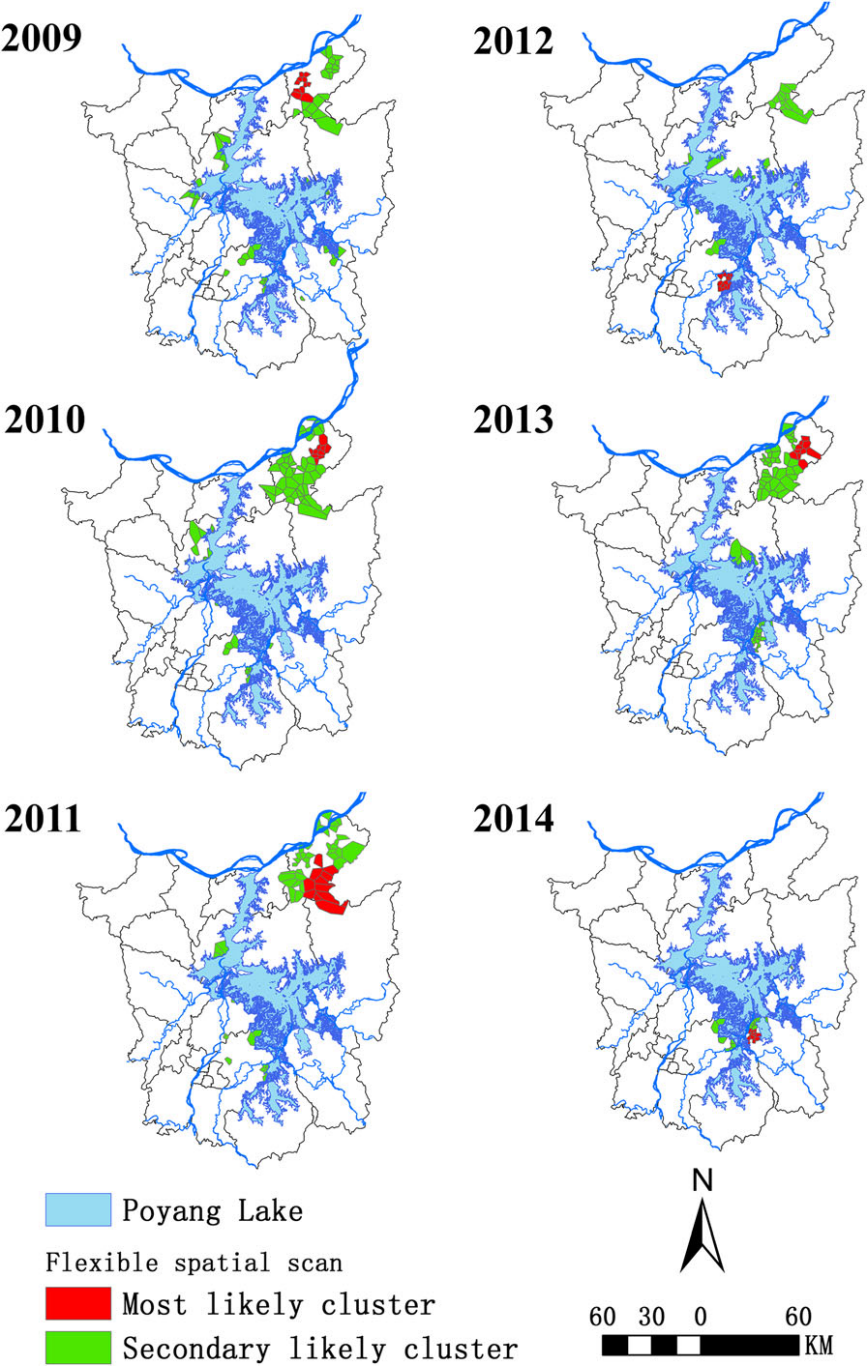
Spatial clusters with a high infection rate of schistosomiasis detected in the period 2009–2014 using the Poisson model by flexible scanning. The cluster length limit was 15. Clusters detected by flexible spatial scan statistic act as irregular shapes and decreased from 14 in 2009 to 4 in 2014 and the number of villages within every most likely cluster fluctuated slightly with narrow range from 10 (in 2010 and 2013) to 13 (in 2013)

Clusters detected by the flexible spatial scan statistic decreased from 14 in 2009 to 4 in 2014. These clusters had irregular contours, but their developmental tendency was identical to that shown by Kulldorff’s method (Fig. 3 and Table 3). Since the maximum cluster length was set at 15, the number of villages within each most likely cluster fluctuated slightly with a narrow range from 10 (in 2010 and 2013) to 13 (in 2013).

**Table 3:** Spatial analysis scanning for clusters with high rates using the discrete Poisson model by flexible spatial scan statistic

Year	No. of clusters	Most likely cluster						
		No. of villages	Maximum distance (km)	No. of cases	Expected no. of cases	RR^a^	LLR^b^	*P*-value
2009	14	11	9.04	364	18.77	19.39	781.85	< 0.001
2010	14	10	10.72	179	15.94	11.23	278.66	< 0.001
2011	12	11	23.83	137	14.88	9.20	189.46	< 0.001
2012	8	13	10.98	36	5.13	7.02	40.92	< 0.001
2013	8	10	15.46	49	4.26	11.50	77.67	< 0.001
2014	4	12	8.48	20	1.40	14.32	36.61	< 0.001

^a^Relative risk

^b^Log likelihood ratio

**Fig. 4:**
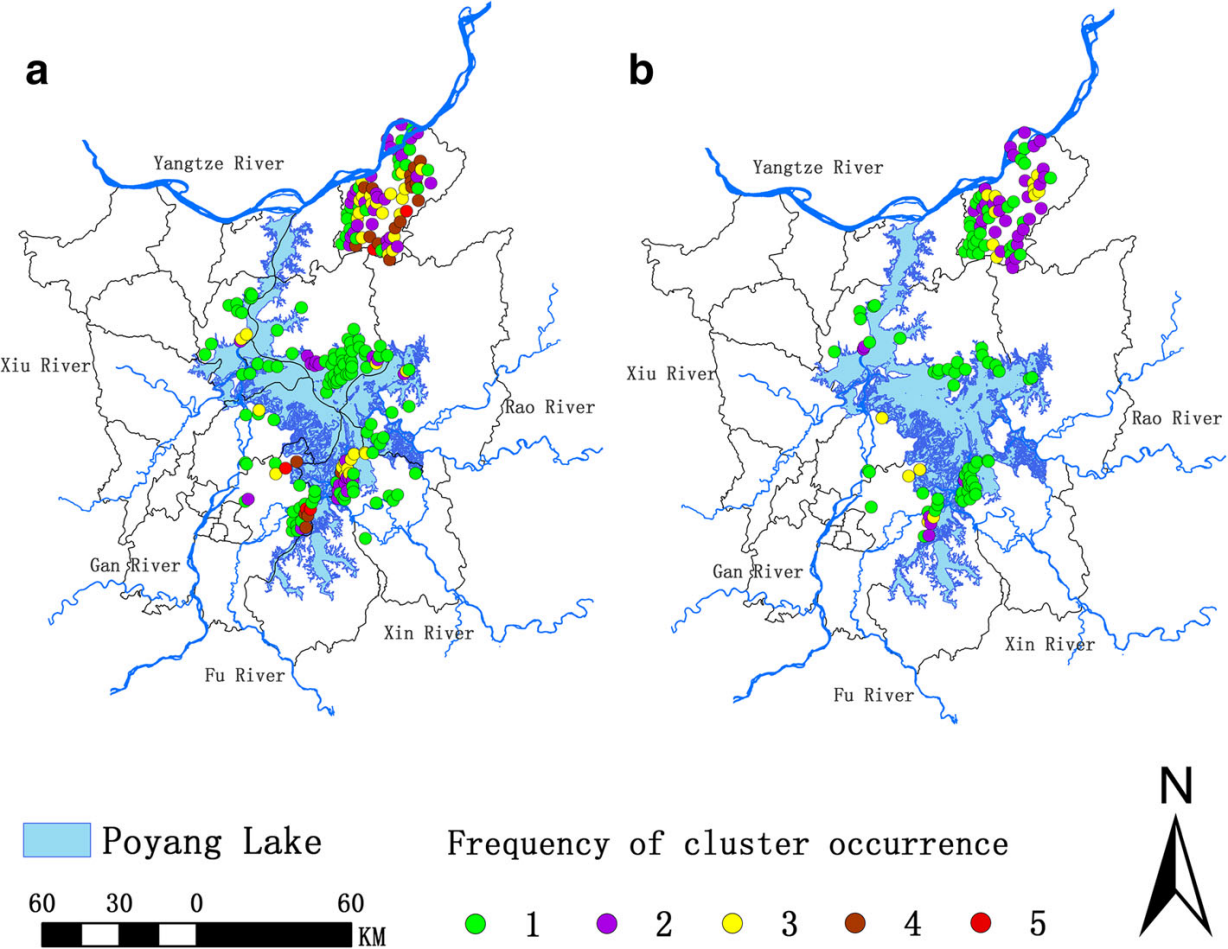
Frequency of cluster occurrence in the period 2009–2014. **a** Frequencies of occurrence of spatial clusters detected by two methods out of the three (Moran’s *I* (Anselin), SaTScan and Flexible scan statistics) each year. **b** Frequencies of occurrence of spatial clusters detected by all three methods. There were 116, 47, 34, 20 high-risk villages (detected by two of the methods) observed one to four times, respectively. For the clusters detected by all three methods, there were 77, 72, 45 strong-evidence high-risk villages detected one to three times, respectively

Figure 4 illustrates the frequency that each village contributed to the clusters during the study period. As seen in Fig. 4a, there were 116, 47, 34, 20 high-risk villages detected contributing to the clusters one to four times during the period 2009 to 2014, respectively. Meanwhile, clusters were found in five villages every year (i.e. five times), three of which were in Nanchang County (Wufeng, Dongyang and Donggfang Villages) and two in Pengze County (Qingfeng and Lufeng Villages).

Strong-evidence high-risk village (Fig. 4b) showed that 77, 72, 45 high-risk villages were detected contributing to the clusters one to three times, respectively. Considering the geographical distribution, high-risk villages including the strong type of evidence that were detected at least once mainly overlap Pengze County and distributed along the Poyang Lake.

### Space-time cluster analysis of infection rate of schistosomiasis in humans

**Fig. 5:**
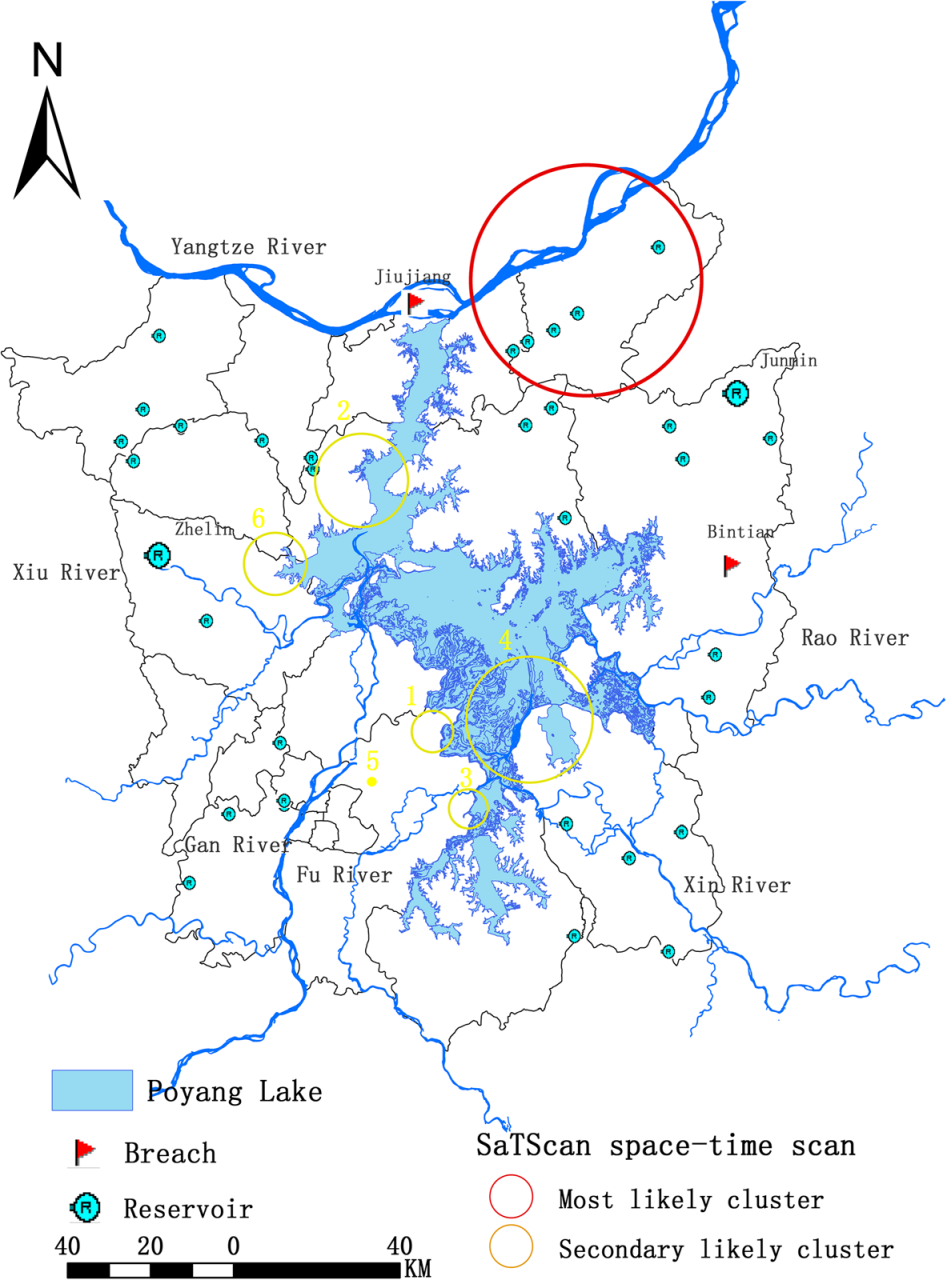
Retrospective space-time analysis of clusters indicating high schistosomiasis infection rate using the discrete Poisson model by SaTScan. Seven clusters (one most likely cluster and six likely secondary clusters) were detected in Poyang Lake Region from 2009 to 2014, which meaning a statistically significant both in space and time in these areas. Considering the temporal clustering pattern, five clusters were observed with the time frame from 2009 to 2011 including the most likely cluster as well as two clusters from 2009 to 2010

As shown in Fig. 5 and Table 4, seven clusters (one most likely cluster and six secondary likely clusters) were detected in Poyang Lake Region from 2009 to 2014 with the biggest log likelihood ratio (LLR) being 3502.96 and the least 31.2 (*P* < 0.001), which indicated a statistical significance both in space and time in these areas. Considering the temporal clustering pattern, five clusters were observed within the 2009 to 2011 time frame including the most likely cluster as well as two clusters in 2009 and 2010.

**Table 4:** Retrospective space-time analysis scanning for clusters with high rates using the discrete Poisson model

Clusters detected		No. of villages in clusters	Time frame	Population	No. of cases	Expected no. of cases	Annual/100,000	Obs/Exp	RR^a^	LLR^b^	*P*-value
Most likely cluster		141	2009–2011	94,430	2203	230.69	827.4	9.55	16.79	3502.96	< 0.001
Secondary likely cluster	1	4	2009–2011	4795	126	12.66	862.1	9.95	10.19	177.52	< 0.001
	2	31	2009–2010	22,343	192	36.44	456.5	5.27	5.45	166.09	< 0.001
	3	8	2009–2011	6904	121	17.33	604.9	6.98	7.14	132.61	< 0.001
	4	50	2009–2011	4,6437	311	112.95	238.5	2.75	2.87	121.18	< 0.001
	5	1	2009–2011	931	24	1.86	1119	12.92	12.98	39.32	< 0.001
	6	13	2009–2010	22,859	86	31.83	234.1	2.73	2.7	31.62	< 0.001

^a^Relative risk

^b^Log likelihood ratio

Regarding the geographical distribution, the most likely cluster with the widest range including a total of 141 endemic villages was mainly concentrated in Pengze County along the River Yangtze (LLR = 3502.96, RR = 16.79). Among these six secondary likely clusters, three major clusters were distributed along the northern side of the Poyang Lake Region. Cluster 4 (Table 4) had an LLR of 121.18 and RR of 2.87 and covered the largest area of 50 endemic villages in Yugan County, generally located at the confluence of River Xin and Poyang Lake. Meanwhile, the cluster 1 (LLR = 177.5, RR = 10.19) and cluster 3 (LLR = 132.61, RR = 7.14), covering 4 and 8 endemic villages, respectively, were mainly distributed in Nanchang County where the River Fu flows. In addition, cluster 2 and cluster 6 were observed along the north-western part of Poyang Lake Basin. Cluster 2 (LLR = 166.09, RR = 5.45) covered 31 endemic villages in Xingzi County while the cluster 6 (LLR = 31.62, RR = 2.7) covered 13 endemic villages across three counties (Yongxiu, De’an, Xingzi), through which River Xiu flows. However, the likely secondary cluster 5 covered only one endemic village, namely Baian Village, which has an LLR of 39.32 and RR of 12.98, located in Nanchang County and the branch of River Fu.

## Discussion

The Poyang Lake Region is widely known as a site for endemic schistosomiasis in China. Schistosomiasis is maintained here due to the suitable climate, geographical environment and the marshland characteristics [12], which are the key factors affecting the transmission of *S. japonicum*. This study analysed the spatial and temporal pattern of schistosomiasis in this area based on annual village-level monitoring data with the objective to provide assistance for future monitor and control.

In spite of an increase in population and the number of endemic villages, this study found that the crude infection rate of schistosomiasis in Poyang Lake Region and the number of high-risk clusters showed a downward trend over time to finally be sustained at a low level. It indicated that there was still a large population at risk but the high-risk areas became more concentrated as time progressed. Therefore, the current control strategy should be increasingly aimed at the hotspots of transmission risk.

The results showed a changing, clustered pattern of the infection rate of human schistosomiasis in Poyang Lake Region. The space-time analysis showed most of the disease clusters concentrated between 2009 and 2011 and then reduced with time, revealing that the schistosomiasis risk sustained at a relatively low level in recent years. From 2011, Jiangxi Province conducted a comprehensive schistosomiasis control strategy based on infectious source control in Poyang Lake Region that included activities based on the slogans, such as “replacing buffalo with machine”, “marshlands isolation and grazing forbidden”, “improving water supply and lavatory”, and improved health education [12]. These efforts probably contributed to a great extent for the reductions of the infection rate observed [25]. Although schistosomiasis control in Poyang Lake Region was clearly successful in recent years, the epidemic is still a severe challenge. The epidemic can persist due to the parasite’s complex life-cycle involving many different definitive mammalian hosts besides humans and cattle and an intermediate snail host with grass-related habitats influenced by the constantly changing environment [26]. Based on the analysis above, we concluded that the identification of hotspots of infection risk should become a vital part in future strategies of precise schistosomiasis control.

The highest number of the most likely clusters, both spatially and temporally as well as the highest frequency of them were detected in Pengze County, through which the River Yangtze flows. On the one hand, we assumed that the hotspots in this area depended on River Yangtze which provides an ideal environment for snail growth and reproduction [27], while on the other, we found that there were five medium-sized water reservoirs within or surrounding this county. The systematic literature review and meta-analysis presented by Steinmann et al. [28] concludes that the development and management of water resources is an important risk factor for schistosomiasis, particularly in Africa. These authors further report that the summary random risk ratio of schistosomiasis due to proximity to dam reservoirs was 2.5 among persons living adjacent to dam or reservoirs [28]. Although, to our knowledge, there is no clear evidence for this association in China, the analysis done by Li and colleagues in 2007 [10] predicted that the Three Gorges Dam would be likely to enlarge the range of the snail habitats and probably result in an increasing number of new schistosomiasis cases in Dongting Lake Region. Therefore, we have reasons to believe that the relatively higher transmission in Pengze County can at least partly be attributed to these reservoirs there. We believe that the construction of these reservoirs could change the ecological environment and the people’s behaviour in a direction favourable for snail habitats and human water contact. Hence, if confirmed by further study, integrated strategies should be adopted to mitigate such negative effects of these reservoirs, including improved water supply, sanitation and, particularly, health education [29, 30].

The other strong evidence clusters were found to be predominantly distributed along Poyang Lake, especially near the inflows of rivers Gan, Fu, Xin and Xiu in Yugan, Nanchang, Yongxiu, Xingzi counties, respectively. These areas were a mixture of lakes, rivers and marshlands along the lake, which appeared alternately in different seasons more frequently than in other regions, providing an ideal environment for snail growth and reproduction [31]. Meanwhile, most of the permanent residents in these endemic counties are farmers and fishermen ensuring increased contact with contaminated water [32]. This finding confirms the link between schistosomiasis and water, thereby providing a reference for public health authorities to assist schistosomiasis control in such high-risk regions by the allocation of resource and additional health care staff. Meanwhile, this study found that several clusters were distributed across counties, indicating that the implementation of schistosomiasis control should not be confined to just one county, but be also conducted in neighbouring counties.

Three cluster detection methods were used in this study to analyse the pattern of schistosomiasis in Poyang Lake Region, which identified different types of clusters, respectively. Local Moran’s *I* measures spatial autocorrelation that determines whether neighbouring areas are more similar than would be expected under the null hypothesis. This method can detect any cluster shape [33] but has serious drawbacks when the sample size varies by area resulting in outliers found in poorly sampled areas [34]. The spatial scan statistic (SaTScan) proposed by Kulldorff defines the potential cluster areas through a circular window [35], so it is difficult to correctly detect noncircular clusters, especially along the rivers including Poyang Lake investigated in this study. The flexible spatial scan statistic is designed to detect a cluster of flexible shape rectifying the problem with SaTScan, but it only works well for small to moderate cluster size (no more than 30) [36]. In this study, the circular clusters provided by this approach would partly overlap the Poyang Lake, thereby inevitably containing also other low-risk areas and even exceed the study scope, while clusters detected by the flexible scan method included endemic villages interconnected by geographical boundaries, thereby showing a higher level of accuracy in the areas along the Poyang Lake and connected rivers. We, therefore, found it more reasonable to combine these three methods, which achieved more precise cluster coverage.

Studies confirm that River Yangtze floods accelerate the spread of *O. hupensis* snails, resulting in the introduction of schistosomiasis into previously non-epidemic areas [15]. It is worth mentioning that Bintian Reservoir, a large reservoir located in Poyang County, broke in June of 2016 because of the drastic rise of the water level this year, causing panic within and around the county. Fortunately, as found by this study, Bintian was far away from all high-transmission clusters, hence we speculated that it might not cause an outbreak of schistosomiasis. However, when the urban dyke of Jiujiang on the southern border of River Yangtze in Jiangxi Province broke in 1988, catastrophic flooding followed causing many cases of schistosomiasis [37]. Indeed, due to the influence of the typhoon “Nepartak” in July 2016, slight breaches of this dyke was found, which is a memento. The lake and marshland regions in the Yangtze River Basin provide an ideal environment for snail growth and reproduction, and the same is true of the Jiujiang Urban Dyke, which is geographically located close to Pengze County. As these areas, including Xingzi, Yugan and Nanchang counties that were within the clusters detected, have been threatened by flooding in recent years and therefore are vulnerable with respect to schistosomiasis transmission, they deserve special attention and reinforcement of dykes and increased surveillance of high-risk counties.

Variables, such as human behaviour, socio-economic level, environment factors (e.g. temperature and precipitation) are of crucial importance in the transmission of schistosomiasis. We plan therefore to include these variables in future models evaluating the risk distribution of schistosomiasis in Poyang Lake Region.

## Conclusions

This study detected areas at high risk for schistosomiasis both in space and time at the village level from 2009 to 2014 in Poyang Lake Region, providing scientific support for improved control activities. The infection rate was dramatically reduced and maintained at a low level in recent years, but large numbers of people are still threatened. The high-risk areas are now more concentrated and mainly distributed at the river inflows Poyang Lake and along River Yangtze in Pengze County. The clusters detected had a tendency of shifting to the South during the six-year study period ending in 2014. Efforts to reduce negative effects should be included when planning and constructing future water projects in Poyang Lake Region.
